# Low-Temperature Emission Dynamics of Methylammonium Lead Bromide Hybrid Perovskite Thin Films at the Sub-Micrometer Scale

**DOI:** 10.3390/nano13162376

**Published:** 2023-08-19

**Authors:** Justine Baronnier, Benoit Mahler, Christophe Dujardin, Julien Houel

**Affiliations:** 1 Université Claude Bernard Lyon 1, Institut Lumière-Matière UMR5306 CNRS, F-69622 Villeurbanne, France; 2 Institut Universitaire de France (IUF), F-75005 Paris, France

**Keywords:** hybrid perovskite, trap, thin films, photoluminescence, microscopy, hyperspectral, lifetime

## Abstract

We study the low-temperature (T = 4.7 K) emission dynamics of a thin film of methylammonium lead bromide (MAPbBr${}_{3}$), prepared via the anti-solvent method. Using intensity-dependent (over 5 decades) hyperspectral microscopy under quasi-resonant (532 nm) continuous wave excitation, we revealed spatial inhomogeneities in the thin film emission. This was drastically different at the band-edge (∼550 nm, sharp peaks) than in the emission tail (∼568 nm, continuum of emission). We are able to observe regions of the film at the micrometer scale where emission is dominated by excitons, in between regions of trap emission. Varying the density of absorbed photons by the MAPbBr${}_{3}$ thin films, two-color fluorescence lifetime imaging microscopy unraveled the emission dynamics: a fast, resolution-limited (∼200 ps) monoexponential tangled with a stretched exponential decay. We associate the first to the relaxation of excitons and the latter to trap emission dynamics. The obtained stretching exponents can be interpreted as the result of a two-dimensional electron diffusion process: Förster resonant transfer mechanism. Furthermore, the non-vanishing fast monoexponential component even in the tail of the MAPbBr${}_{3}$ emission indicates the subsistence of localized excitons. Finally, we estimate the density of traps in MAPbBr${}_{3}$ thin films prepared using the anti-solvent method at *n*∼10${}^{17}$ cm${}^{-3}$.

## 1. Introduction

Halide-based, hybrid organic–inorganic, perovskite materials have attracted the world’s attention since 2012 through the demonstration of a low-cost solar cell approaching energy conversion records [1,2,3]. Since then, halide perovskite materials have successfully made their ways through multiple arrays of opto-electronics: solar cells [4], LEDs [5], lasers [6], photodetectors [7], solar concentrators [8], mechanical energy harvesting [9] or scintillation devices [10,11]. While methylammonium lead iodide (MAPI) has been arguably the most successful halide perovskite so far for application in solar cells [12,13], methylammonium lead bromide (MAPbBr${}_{3}$) thin films or single crystals find applications in LEDs, scintillation and photodetector devices [10,14,15]. The dynamics and origin of their emission have thus been the center of several studies and are still highly debated subjects [16,17,18,19,20,21,22,23,24,25]. In order to improve device quality, it is also important to understand the spatial dependence of their emission, i.e., performing not only ensemble measurements, but also spatially resolved experiments, at the micrometer scale [26,27]. Indeed, while it was originally believed that the grain boundaries in polycrystalline thin films were causing no harm to device efficiency [28,29], it has been demonstrated otherwise [26,27,30]. Concerning the spatial characterization of the emission, much effort has been focused on the spatial inhomogeneity of the emission of MAPI [26,30,31], but information is more sparse for MAPbBr${}_{3}$ thin films [27,32,33]. Usually, only two of the three following information sources are available: spatial, spectral and temporal. It is, however, efficient to identify the emitting entities with the spectral information and the corresponding emission dynamic mechanism with fast temporal information [16,17,20] and couple it to spatial information [26,30]. Concerning the emission dynamics of MAPbBr${}_{3}$ thin films (contrary to MAPbBr${}_{3}$ single crystals [16,34,35,36]), most of the studies were performed at room temperature, where the thermal energy is large enough to ionize the shallow defects and observe free carrier relaxation [19]. There have been very few studies at low temperature, down to T = 10 K, where the shallow defect recombination dynamics are modified and free carrier relaxation is not expected [17,20,25,37,38,39,40,41]. Low-temperature experiments enable one to probe thin film dynamics (and more specifically MAPbBr${}_{3}$ in our case) without being hampered by the following: strong exciton–phonon interactions that take place at room temperature and create strong overlap between the emission peaks [25], organic cations’ rotational motion [17], or free carrier emission [38,41]. It is thus a unique tool that allows one to focus solely on the excitons’ and the traps’ emissions. Furthermore, while most devices based on MAPbBr${}_{3}$ (and especially LEDs) operate at room temperature, the differences between room- and low-temperature emissions [39] may lead to a better understanding of some physical aspects that govern the performances of LEDs [25]. Moreover, it is now well-known that MAPbBr${}_{3}$ undergoes phase transitions as the temperature is lowered [17,38,42] and that working at cryogenic temperatures unlocks the possibility to probe the physics of the orthorombic crystalline phase, which is not accessible at room temperature. Finally, it is important to study the low-temperature response of hybrid perovskite thin films in the framework of new applications where they can serve as a passive medium for quantum optics applications [43]. Hyperspectral and fluorescence lifetime imaging microscopy (FLIM) performed at low temperature are interesting techniques to access altogether spectral, temporal and spatial information at the sub-micrometer scale and unravel the aforementioned properties. In this paper, we present a study which joins the four properties: spatial, spectral or temporal are observed at low temperature (T = 4.7 K) for the emission of MAPbBr${}_{3}$ thin films prepared via the anti-solvent method. The first part is dedicated to continuous wave (CW) experiments performed with an excitation quasi-resonant with the MAPbBr${}_{3}$ band-edge emission. Intensity-dependent hyperspectral imaging enables us to locate spatially and spectrally the emissions of excitons and defects. In the second part, two-color FLIM experiments are presented, which allow us to estimate the density of defects in the MAPbBr${}_{3}$ thin film, as well as the main process of recombination through these traps and its evolution with the density of absorbed photons. Our work permits us to image and discriminate for the first time at T = 4 K the regions of emission arising from the excitons or the traps at the micrometer scale, and subsequently propose a model that explains the trap emission via a Förster resonant transfer of diffusing electrons recombining with static holes. We find that, contrary to another report, the excitons’ dynamics are represented by a fast exponential and not a long-tail process [17]. These findings can have implications in improving the performances of devices based on light harvesting, light emission and future low-temperature applications of the hybrid perovskite thin films.

## 2. Materials and Methods

### 2.1. Methylamonium Lead Bromide Thin Film Preparation

The MAPbBr${}_{3}$ thin film was prepared as described in Ref. [43]. Briefly, 112 mg of methylamonium bromide (MABr, 98%, Sigma Aldrich) powder and 367 mg of lead bromide (99.999%, perovskite grade, Sigma Aldrich) were dissolved in 2 mL of N-N-Dimethylformamide (anhydrous DMF 99.8%, Sigma Aldrich) leading to a 0.5 M solution of MAPbBr${}_{3}$ precursor. Then, 100 μL of that solution was drop-cast on a microscope coverslip (BK7) at 5000 rpm for 30 s. Five seconds after starting the spin-coater, 500 μL of chlorobenzene (anhydrous 99.8%, Sigma Aldrich) was injected to enforce homogeneous crystallization of the MAPbBr${}_{3}$ thin film. The result was a highly oriented, transparent, crystalline thin film (see Supplementary Materials Section S1) with a thickness of 90 nm measured with an atomic force microscope (AFM) [43].

### 2.2. Low-Temperature Confocal Microscopy

The setup is described in detail in the Supplementary Materials, Section S2. The sample was placed on three-axis steppers and scanners in a home-built confocal microscope coupled to a helium recycling cryostat. The system allows one to perform experiments at T = 4.7 K at the sample location. The excitation was provided by either a picosecond pulsed laser emitting at 445 nm, or a CW laser emitting at 532 nm. The excitation was sent to a half-waveplate and a polarizer, then entered a polarizing beam-splitter (PBS) which reflected the laser beam down to a quarter-waveplate and finally reached the microscope objective (which was at T = 4.7 K) as circularly polarized light. The microscope objective focused the beam onto the sample and collected the luminescence. The collected PL was redirected to the PBS and was coupled into a single mode fiber and sent to a 300 mm focal length spectrometer and a Handbury Brown and Twiss interferomter (HBT) via a 2 × 2 fiber coupler.

### 2.3. Hyperspectral Microscopy

For hyperspectral imaging measurements, the sample was excited with a 532 nm CW laser. The sample was raster-scanned through the laser beam by moving the xy low-temperature scanner. The scanning range was 9 × 9 μm${}^{2}$ at T = 4.7 K. Light collected from the microscope was sent to a 303 mm focal length monochromator and dispersed by a 600 line/mm grating. Spectral information was recorded by an emCCD camera cooled down at minus 80 ${}^{\circ }$C. The system spectral and spatial resolution were 0.24 nm and 500 nm, respectively.

### 2.4. Two-Color Fluorescence Lifetime Imaging Microscopy

For FLIM measurements, the sample was excited with a picosecond pulsed laser at 445 nm. The sample was raster-scanned through the laser beam by moving the xy low-temperature scanner. The scanning range was 9 × 9 μm${}^{2}$ at T = 4.7 K. Light collected from the microscope was sent to an HBT interferometer consisting of two single-photon avalanche photodiode (SPAD) detectors (Micron Photon Devices, PDM) coupled by a 50:50 R/T beamsplitter. In front of the first SPAD (DET1), we have placed a longpass filter at 550 nm (Thorlabs, FELH0550) and a bandpass filter centered at 550 nm (Edmund optics), generating a 5 nm detection window ranging from 550 to 555 nm. In front of the second detector (DET2), a bandpass filter centered at 568 nm with 10 nm bandwidth was placed, creating the second detection window at 568 ± 5 nm. Both detectors recorded the PL signal from the microscope simultaneously, allowing for two-color FLIM experiments. The SPADs were connected to a multi-channel photon counting electronics with 80 ps time bin. The system timing and spatial resolution before reconvolution-based fitting were 240 ps and 700 nm, respectively. Up to four repetition rates were used: 5, 1, 0.5 and 0.1 MHz, with an integration time per pixel of 5 s for the first two, and 10 s for the last two.

### 2.5. Fitting the Fluorescence Lifetime Imaging Experiments

Each FLIM image contains 5000 decay curves, all of which were fitted using the “curve fit” algorithm from the SciPy library in Python. In order to keep the free parameters of the fit to a minimum, the photon background was estimated for every curve to the median value of the first 10 points in the decay curve. We used re-convolution with the IRF (see Supplementary Materials, Figure S4) of our system to obtain more sensible results for the fast component.

## 3. Results

### 3.1. Hyperspectral Microscopy Under Continuous, Quasi-Resonant Excitation

#### 3.1.1. Ensemble Measurements

**Figure 1:**
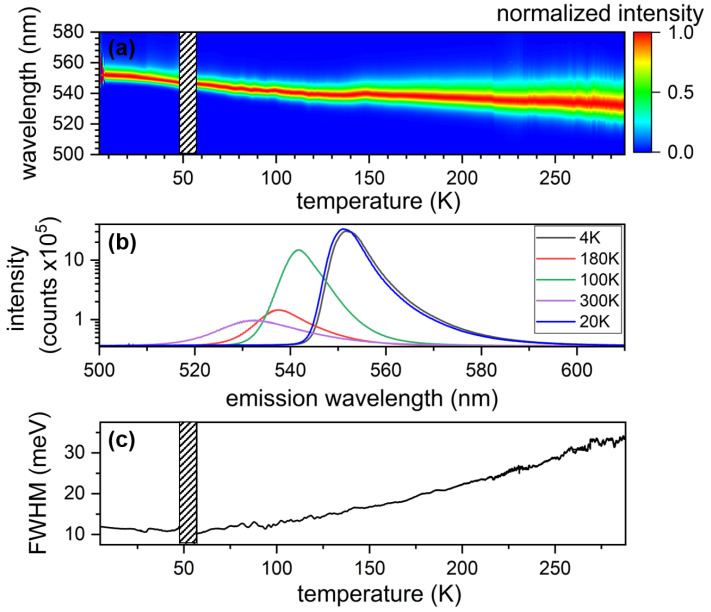
(**a**) Photoluminescence signal of the MAPbBr${}_{3}$ thin film as a colorscale as a function of temperature. (**b**) Logarithm of the emission spectrum of the MAPbBr${}_{3}$ thin film for five different temperatures: 300 (violet), 180 (red), 100 (green), 20 (blue) and 4 K (black). (**c**) Full width at half maximum of the emission of the MAPbBr${}_{3}$ thin film as a function of temperature. The blank column in (**a**,**c**) are data removed from the processing.

We present in Figure 1 ensemble characterizations of a MAPbBr${}_{3}$ thin film during the sample cool-down from T = 300 to 4.7 K. Figure 1a presents the evolution of the normalized emission of the MAPbBr${}_{3}$ thin film under an excitation of $\lambda_{ext}$ = 445 nm, as a function of time. The emission spectrum has its maximum shifting from $\lambda_{max}=532$ nm at 300 K to 552 nm at 4.7 K. This is in accordance with previous results in the literature [17]. We note a discrete change in the emission wavelength at a temperature of T∼150 K, which might be the subtle sign of the MAPbBr${}_{3}$ thin film transition from the tetragonal to orthorombic phase [17,38,42]. Figure 1b presents five examples of spectra recorded at five different temperatures: 300 K (violet), 180 K (red), 100 K (green), 20 K (blue) and 4.7 K (black). Intensities are presented in log scale. At high temperature, the spectrum is described by two peaks [16,17,43], while at low temperature there is the apparition of an emission tail at longer wavelength. At room temperature, the first high-energy peak at 532 nm is usually attributed to excitons [17,38,40], while the origin of the low-energy peak is still debated [17]. Figure 1c presents the evolution of the linewidth of the emission of the MAPbBr${}_{3}$ thin film as a function of temperature. It varies from 35 meV at 300 K to 12 meV at 4.7 K. We note that we do not see a signature of the phase transition in the emission linewidth, while we see a hint of it on the emission wavelength.

#### 3.1.2. Hyperspectral Microscopy

**Figure 2:**
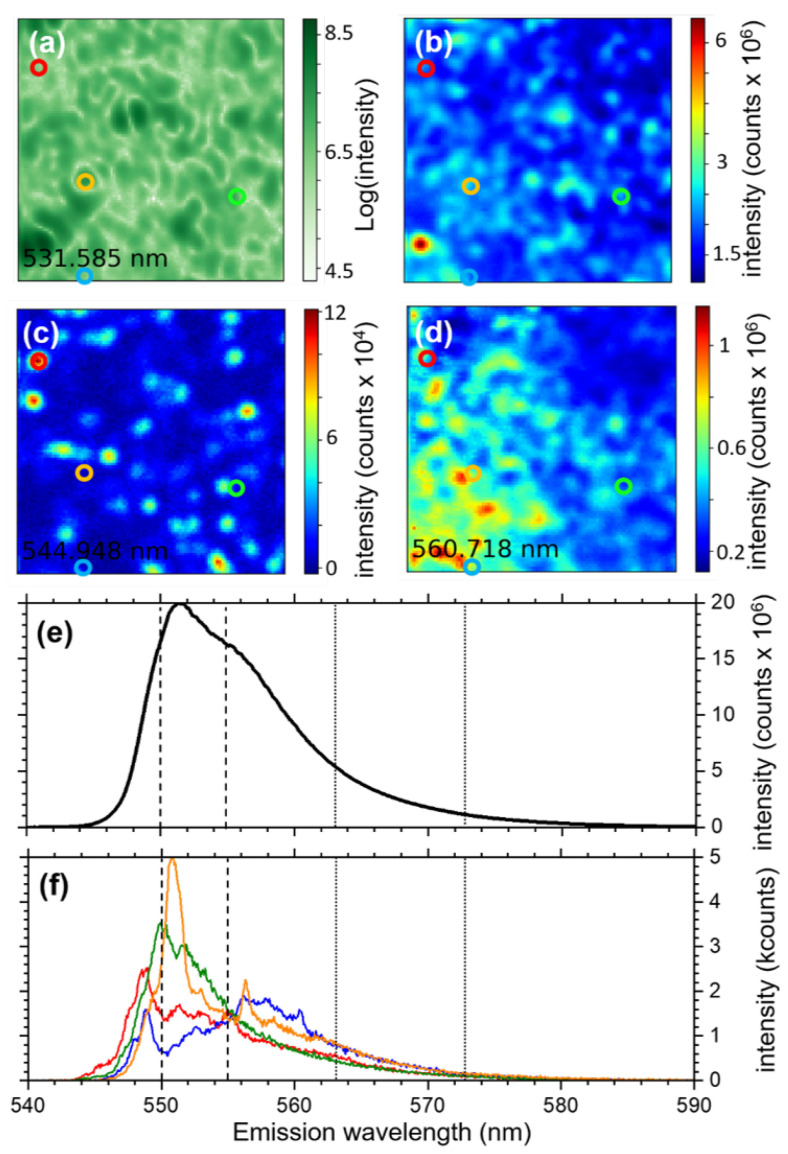
Results from a 9 × 9 μm${}^{2}$, 100 × 100 pixel hyperspectral imaging of Zone 1 in the MAPbBr${}_{3}$ thin film recorded for an excitation intensity 100 nW/μm${}^{2}$. (**a**) Signal obtained at at 531.6 ± 0.5 nm in Zone 1 (wavelength of the excitation laser). (**b**) Image of Zone 1 obtained by integrating the emission spectra in every pixel of the hyperspectral image between 540 and 580 nm. (**c,d**) Emission mapping of Zone 1 at 545 ± 0.5 nm (**c**) and 561 ± 0.5 nm (**d**). (**e**) Spectrum obtained by summing all the spectra in every pixel from (**b**). (**f**) Emission spectra corresponding to the pixels in the center of the similarly colored circles in (**a**–**d**).

At T = 4.7 K, we have recorded a hyperspectral, 9 × 9 μm${}^{2}$ image of Zone 1, at an excitation wavelength of 532 nm and an intensity of 100 nW/μm${}^{2}$, where each spatial pixel encompasses a spectrum ranging from 500 to 600 nm. Results obtained for different wavelength ranges are presented in Figure 2a–d. Figure 2a shows the signal recorded around 532 nm, i.e., the excitation laser wavelength. It is thus not an image of the MAPbBr${}_{3}$ emission, but a confocal retro-reflectivity image of the sample surface. It gives us information on the topography of the sample. We observe that the thin film is locally structured with polycrystalline domains of ∼1 μm wide surrounded by the grain boundaries. This is in accordance with SEM images of our thin films [43]. Summing the spectra in every pixel for 540 nm ≤λ≤580 nm, we obtain the image in Figure 2b, which represents a confocal PL image of Zone 1. Except from a high-intensity spot in the bottom-left of the image, the PL signal from the thin film is rather homogeneous, varying by less than a factor of 2 over the whole image. We notice a moderate correspondence between the two images (topography and full-integrated PL) corresponding to a Peasron’s *r* correlation coefficient between the two images of r=0.41 (*r* takes any value between −1 and 1, *r* = −1 being a complete anticorrelation of the data, *r* = 0 the absence of correlation and *r* = 1 a complete correlation). We present in Figure 2c,d two images obtained by integrating the spectra in every pixel with a bandwidth of 0.8 nm at 545 nm (Figure 2c) and 561 nm (Figure 2d). The image in Figure 2c seems drastically different from the one with fully integrated spectra in Figure 2b. The corresponding Pearson’s *r* between (c) and (b) reads at r=−0.01, i.e., no observable correlation. Figure 2c is structured with resolution-limited (∼500 nm), low-density emission spots, which have a strong signal-to-background dynamic of about 100. There are approximately 30 spots in the 81 μm${}^{2}$ of Figure 2c, i.e., less than 0.5 spot per micron-squared. These emission spots appear to be randomly distributed over the Zone 1 surface. In contrast, Figure 2d (signal at 561 nm) exhibits a large collection of emission spots, leading to a continuous signal and a smaller dynamic in the image, with a max-to-min reading at about 6. There is a clear asymmetry in the emission signal, from low at the top-right, to high in the bottom-left. In this case, it more closely resembles Figure 2b, the fully integrated spectra image. Indeed, the Pearson’s *r* between those two images ((b) and (d)) is r=0.43, i.e., a moderate correlation. Finally, the Pearson’s *r* between the high- (Figure 2c) and low-energy (Figure 2d) seats is r=−0.12, denoting a weak anti-correlation between the two signals. Figure 2a–d thus leads us to conclude that the emission at low temperature of a MAPbBr${}_{3}$ thin film is strongly spatially and wavelength dependent with an emission spectrum extremely richer than what has been measured before on ensemble measurements [17,40]. Figure 2e presents the sum of all spectra from all the pixels in Figure 2b. We obtain a continuous curve, with a strong asymmetric tail to the low-energy areaand a double-peak, whose maxima are located at 552 and 555 nm. Figure 2f presents spectra from single pixels located by colored-circle on the four images in Figure 2a–d. The colors of the spectra correspond to the pixels in the center of the similarly colored circles. Surprisingly, every pixel exhibits a different spectrum. Moreover, while the spectra are all quantitatively different, they all exhibit a structure with multi-peaks at high energy and a continuous tail at low energy. These narrow peaks (<1 nm) are located in the spectral region ∼545–560 nm. While in the region ∼545–550 nm the peaks seem to be isolated, those located in the region ∼550–560 nm are rising on top of what appears to be a continuous background.

#### 3.1.3. Power Dependence

**Figure 3:**
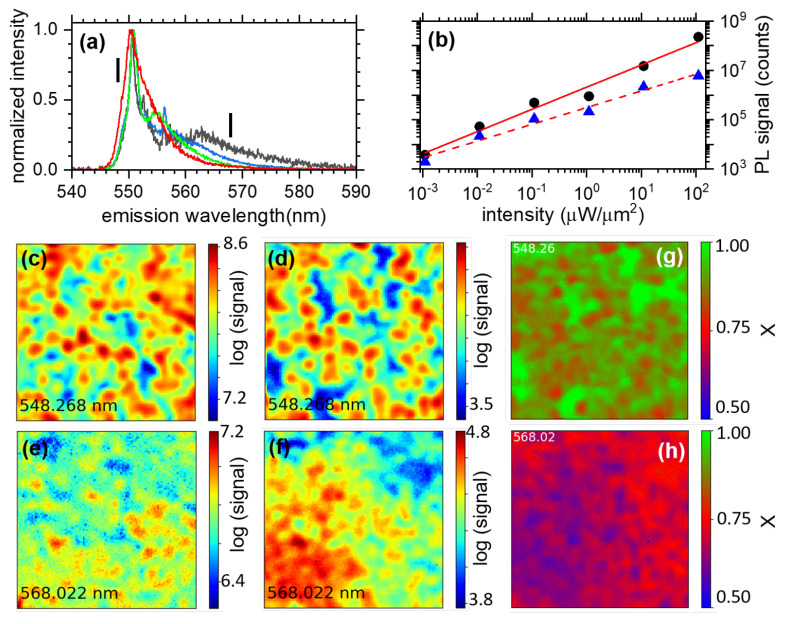
(**a**) Normalized emission spectra of the pixel in the center of the orange circle of Figure 2 for 4 four different excitation intensities (at 532 nm): 1 nW/μm${}^{2}$ (black), 100 nW/μm${}^{2}$ (blue), 10 μW/μm${}^{2}$ (green) and 100 μW/μm${}^{2}$ (red). (**b**) Integrated PL signal from pixel (84,81) as a function of excitation intensity over 5 decades, in a log-log plot. Full dark circles are data at 549 nm emission wavelength, while blue triangles represent data at 568 nm. Red solid and dashed lines are linearly fit to the data following Equation (1), with X = 0.91 ± 0.06 (solid line) and X = 0.67 ± 0.04 (dashed line). The two wavelengths at which the curves were extracted are indicated by two small black segments in (**a**). (**c**–**f**) Examples of images extracted from a 9 × 9 μm${}^{2}$, 100 × 100 pixel hyperspectral imaging of Zone 1 in the MAPbBr${}_{3}$ thin film for two different excitation intensities and emission wavelengths. (**c**,**d**): Spatially resolved emission signal of the MAPbBr${}_{3}$ thin film at wavelength 548.3 ± 0.40 nm for intensities of 100 μW/μm${}^{2}$ (**c**) and 10 nW/μm${}^{2}$ (**d**). (**e**,**f**) The same intensities used in (**c**,**d**) but for emission spectra integrated over 568 ± 0.40 nm. (**g**,**h**): Image of 9 × 9 μm${}^{2}$, 100 × 100 pixel representing *X* as a colorscale obtained from the fitting procedure (following Equation (1) performed for every pixel of Zone 1 for 6 excitation intensities ranging over 5 decades from 100 μW/μm${}^{2}$ down to 1 nW/μm${}^{2}$.

We present in Figure 3a an intensity dependence of the shape of the spectrum from the pixel in the center of the orange circle from Figure 2. The intensity varies over 5 orders of magnitude from 1 nW/μm${}^{2}$ (black line) to 100 μW/μm${}^{2}$ (red line). In between, we find the spectra recorded at 100 nW/μm${}^{2}$ (blue line) and 10 μW/μm${}^{2}$ (green line). The spectra are normalized to their respective maximum. The intensity of the low-energy part of the spectrum decreases relative to the band-edge emission. In order to characterize further this behavior, we have recorded hyperspectral images of Zone 1 for those six excitation intensities covering 5 decades, from to 100 μW/μm${}^{2}$ down to 1 nW/μm${}^{2}$ at an excitation wavelength of $\lambda_{exc}$ = 532 nm. We present in Figure 3b an example of the corresponding power dependence of the emission obtained on a single pixel from Zone 1 for two different emission wavelengths, namely, 549 nm (black dots) and 568 nm (blue triangles). The two curves are plotted in a log-log manner and their behavior can be well described by a linear interpolation, i.e., a power law in linear coordinates. We fit those two set of data with the following equation:

(1) $$S\left(I\right)=A\times I^{X}$$

where *S*, *A*, *I* and *X* are the PL signal, the amplitude, the excitation intensity and the power law exponent, respectively. We find X= 0.91 and 0.67 at 549 and 569 nm, respectively, indicating a quasi-linear increase in the PL of the MAPbBr${}_{3}$ thin film at 549 nm (characteristic of exciton emissions [44,45]), but a strongly sub-linear behavior at 568 nm, suggesting a saturation effect (characteristic of a finite number of traps [17,45]). Extending this analysis to the entire Zone 1, we present in Figure 3c–f examples of the obtained hyperspectral images integrated over different spectral regions, with the same bandwidth of 0.8 nm. The full hyperspectral data are presented in the Supplementary Materials in the form of a series of seven videos (one for every decade of powers and one for the final image of the spatial distribution of *X*), where one frame represents 0.08 nm: it highlights the strong spectral and spatial inhomogeneity of the MAPbBr${}_{3}$ thin films, which cannot be fully presented in the manuscript. In Figure 3c,d, the spectra are integrated over 548.3 ± 0.4 nm for excitation intensities of 100 μW/μm${}^{2}$ and 10 nW/μm${}^{2}$, respectively. Figure 3e,f was obtained by integrating the spectra over 568 ± 0.4 nm for the same intensities as (c) and (d), respectively. All contour plots are presented with intensity in log scale. A more complete figure is presented in the Supplementary Materials, Figure S3. More precisely, Figure S3d,f presents the imaging for spectra integrated over 561 ± 0.4 nm. The images in Figure 3c,d are highly comparable, pointing to a spatially homogeneous evolution of the signal at that emission wavelength. Indeed, while the PL signal is inhomogeneously distributed over the surface, its evolution as a function of intensity at the band-edge proves to be homogeneous. However, as the emission wavelength increases, the image changes from low to high intensities, indicating non-linearity in the evolution of its spatial inhomogeneity: as the power is increased, the image becomes more and more homogeneous, indicating that the emission from some regions in the image are experiencing a saturation that is not experienced for images at the band-edge. We present in Figure 3g,h and in Figure S3k, 100 × 100 pixel images representing the spatial distribution of *X* as a colorscale over Zone 1, for emission wavelengths 548 nm (Figure 3g), 561 nm (Figure S3k) and 568 nm (Figure 3h). On a general level, we see that *X* decreases on average from high to low emission energy. We find the following average values: $<X_{548}>$ = 0.9 for the image at 548 nm, $<X_{561}>$ = 0.76 for the image at 561 nm and $<X_{568}>$ = 0.68. Looking at the details, we find that for the image at 548 nm (Figure 3g) the signals from the inter-spaces between spots of high signal behave linearly with the excitation intensity (green color represents *X*∼1), while the spots themselves behave sub-linearly, with a red color, indicating *X*∼0.75. At an emission wavelength close to 560 nm (Figure S3k), the power law exponent *X* reveals to be homogeneous for the whole image around *X*∼0.75. While we can conclude for Figure 3g that the green regions result from the emission of linear entities with excitation power, the equivalent straight conclusion about only sub-linear entities emitting in Figure S3k and Figure 3h cannot easily be drawn. Indeed, the emission could be the superposition of linearly behaving entities and sub-linear ones, leading to X< 1. To support that assumption, one can still observe faint green regions over Figure S3k. Finally, at even larger emission wavelengths, 568 nm (Figure 3h) in the emission tail, we find that the region with the highest signal behaves similarly to the square root of the excitation intensity with *X*∼0.5 (blue regions), while the lower-signal regions behave like those at 561 nm and on the spots of high signal at the band-edge, i.e., with *X*∼0.75. From these power dependence experiments, we can conclude that part of the band-edge emission behaves linearly with the excitation intensity, as should the exciton. Whereas, as the wavelength increases, most of the emission in the image behaves sub-linearly, even similar to the square root of the intensity for the regions of Zone 1 at 568 nm. At the band-edge, we are able to locate spatially the micrometer-scale regions over which the excitons’ emission dominated the signal. We conclude from the latter results that the emission at longer wavelength than the band-edge is a subtle mix between excitons and defects; however, these CW experiments do not permit us to unwrap the mixing, its evolution within the excitation intensity, nor to determine the emission mechanism at the tail of the emission.

### 3.2. Fluorescence Lifetime Imaging Microscopy Under Pulsed, Non-Resonant Excitation

In order to understand in more detail this behavior, we have performed time-resolved measurements, which will give us access to the emission dynamics of excitons and traps. We present in the following section results from FLIM experiments performed on a 9 × 9 μm${}^{2}$, 50 × 50 pixel area (Zone 2). To perform these experiments, part of the signal is directed to a Handbury Brown and Twiss intererometer (HBT). In front of the first detector (DET1) the detection window ranges from 550 to 555 nm, i.e., close to the band-edge. In front of the second detector (DET2) the detection window is 568 ± 5 nm. The bandwidth of DET1 and DET2 are represented as dashed and dotted lines, respectively, in Figure 2e,f. Details of the setup are presented in the Supplementary Materials, Sections S2 and S4.

#### 3.2.1. Exciton- and Trap-Related Emission Dynamics

**Figure 4:**
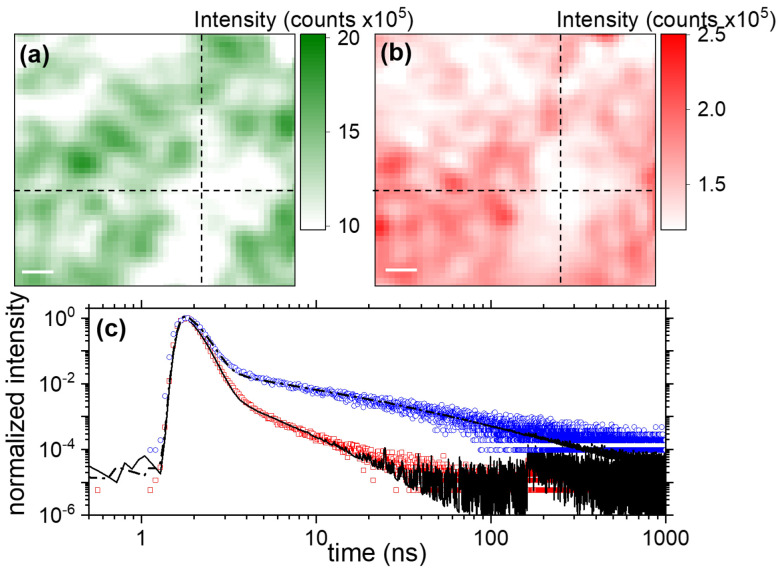
(**a**,**b**) 9 × 9 μm${}^{2}$, 50 × 50 pixel, PL microscopy obtained at an excitation wavelength λ = 445 nm, a repetition rate $f_{rep}$ = 500 kHz and an intensity of 40 nW/μm${}^{2}$. The PL signal is filtered for DET1 at 550–555 nm (**a**) and for DET2 at 568 ± 5 nm (**b**). The PL intensity is presented as a linear colorscale. Integration time per pixel was 10 s. (**c**) Decay curves extracted from the pixel at the intersection of the two black dashed lines in (**a**,**b**). The data are presented in log-log scale. The fits performed following Equation (2) are shown as solid black (DET1) and dashed dot line (DET2). The fitting parameters for DET1 are A${}_{1}$ = 0.71, $\gamma_{1}$ = 5.5 ns${}^{-1}$, A${}_{2}$ = 0.054 $\gamma_{2}$ = 50 ns${}^{-1}$ and β = 0.32. The fitting parameters for DET2 are A${}_{1}$ = 0.71, $\gamma_{1}$ = 4.2 ns${}^{-1}$, A${}_{2}$ = 0.071 $\gamma_{2}$ = 50 ns${}^{-1}$ and β = 0.22. The signal increase at time 160 ns, with an amplitude 4 orders of magnitude smaller the maximum signal is an electronic feature of our setup and is fully accounted for by the IRF (see Supplementary Materials, Figure S4). Integration time per pixel was 10 s.

**Figure 5:**
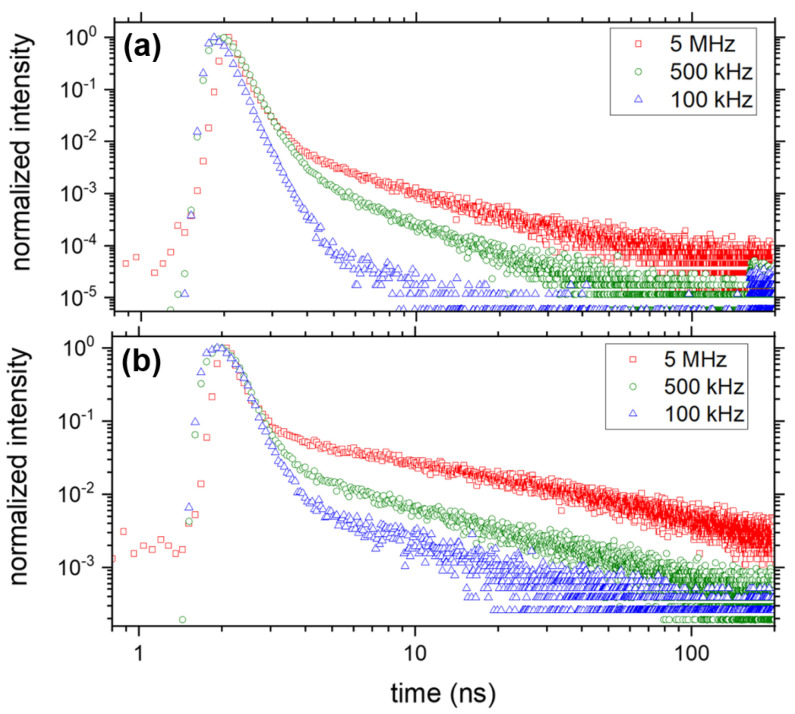
Decay curves obtained on the same pixel as Figure 4 for different repetition rates (equivalent $n_{i}$) at <P> = 240 nW/μm${}^{2}$ average excitation intensities: 5 MHz (red), 500 kHz (green) and 100 kHz (blue) for DET1 and signal filtered between 550 and 555 nm (**a**), and for DET2 filtered at 568 ± 5 nm (**b**). Integration times were 5 and 10 s for 5 MHz and 0.5/0.1 MHz, respectively.

**Figure 6:**
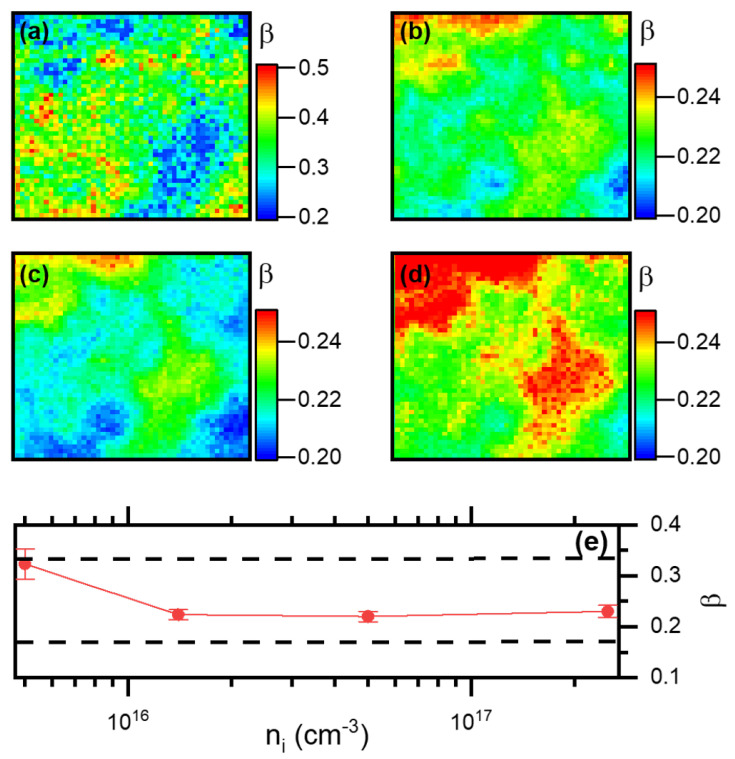
(**a**–**d**): A 9 × 9 μm${}^{2}$, 50 × 50 pixel image of Zone 2 where a pixel takes the value of the fitting parameter β from Equation (2) represented as a colorscale, for $n_{5}$ (**a**), $n_{1}$ (**b**), $n_{0.5}$ (**c**) and $n_{0.1}$ (**d**). (**e**): Average value of β calculated over the complete images (**a**–**d**) for different values of $n_{i}$. The dashed lines represent value of β for 2D (top line) and 1D Förster direct transfer (bottom line) when all the defect distribution is available for diffusion (no truncation to nearest neighbor).

We present in Figure 4a,b the PL intensity map of Zone 2 from DET1 ((a), 550–555 nm) and DET2 ((b), 568 ± 5 nm) obtained at a repetition rate of 500 kHz. Because of the larger bandwidth of the images in (a) and (b), compared to the images of Zone 1 (shown with 0.8 nm bandwidth), the visual correspondence between (a) and (b) appears to be larger, while taken at center wavelength 16 nm away. This is confirmed by the Pearson’s *r* found to be r=0.65, indicating a positive correlation between the two images. The correlation is, however, only partial (r<1), highlighting the difference induced by the two spectral windows used to record the images. The signal over the surface is rather homogeneous since, for both images, the full-scale dynamic of the signal is close to 2. Figure 4c presents two examples of decay curves obtained for the same pixel in Zone 2, indicated by a dashed cross in both images in Figure 4a,b. Results are presented in log-log scale to capture visually the long- as well as the short-time dynamics. The red circles in Figure 4c correspond to the lifetime close to the band-edge around ∼550 nm (Figure 4a), and the blue circles the decay around 568 nm (Figure 4b). The curves are normalized to their respective maximum. Both curves exhibit a similar qualitative behavior: we can identify a fast and a slow process. However, the amplitude of the slow component appears to be largely diminished at wavelengths closer to the band-edge (500–550 nm) compared to wavelengths identified to be defect-related with the CW experiments (568 nm). From that correspondence, we assign the fast component to the exciton dynamics, and the stretched exponential to trap emission. While it is difficult to assign a precise behavior at first sight to the fast component, which is tangled with the instrument response function (IRF, see Supplementary Materials Figure S4), it is straightforward to conclude with the log-log plot that the slow process is neither mono- or bi-exponential but buries a complex, multi-exponential structure. Our different fitting attempts eliminated a strict power law to describe the behavior of the long-lived component, and we found that a stretched exponential was a better fit for both channels. Stretched exponential dynamics in hybrid perovskite have been observed in thin films of MAPbCl${}_{x}$I${}_{3-x}$ [26,46,47] and in MAPbBr${}_{3}$ [17,45]. It is not surprising to observe complex decay dynamics in the emission tail (usually denoted an Urbach tail) since it is acknowledged that its emission is partially driven by intrinsic disorder and defects [19]. The association of those two processes, one fast mono-exponential in addition to a stretched exponential, was used to fit the data as follows:

(2) $$I\left(t\right)=y_{0}+A_{1}\times e^{-\gamma_{1}\times t}+A_{2}\times e^{-{\left(\gamma_{2}\times t\right)}^{\beta }}$$

where *y*${}_{0}$, *A*${}_{1}$, *A*${}_{2}$, $\gamma_{1}$, $\gamma_{2}$ and β are the background, the amplitudes of the fast and stretched components, the decay rate of the fast and stretched component and the stretching exponent. We obtained the results presented in Figure 4c where the fits are shown as black lines. For the two fits, we find $\gamma_{1}∼\;$4–5 ns${}^{-1}$ (corresponding to a decay time of the fast process equivalent to $\tau_{1}$ = 1/$\gamma {}_{1}∼$ 200–250 ps). Similar fast decay times are observed in CsPbBr${}_{3}$ perovskite quantum dots [48] at T = 4 K or in CsPbBr${}_{3}$ single crystals [16]. Regarding the trap-related relaxation, we find β = 0.32 and 0.22 for 550 and 568 nm. Stretched exponential dynamics can be the mathematical description of a diffusion process [17,47,49] and do not describe typical donor–acceptor static relaxation [17,49], which resembles a stretched exponential if one is not careful enough to record the full dynamics over several orders of magnitude in the time and signal axes [17]. On the other hand, electrons diffusing over randomly distributed traps, and recombining with static holes on acceptors can give rise to a stretched exponential [49]. For example, the relaxation function probability ϕ(t) of an electron diffusing through a collection of randomly distributed traps via the Förster direct-transfer model, based on a $R^{-6}$ interaction (where *R* is the distance to the trap), is written as [49]:

(3) $$\phi \left(t\right)=\exp(-{\left(\gamma \times t\right)}^{\beta })$$

where γ is the relaxation rate and β=D/6 for a process in *D* dimensions. Thus, we expect β to vary from 0.5 to 0.17 as the dimension of the diffusion goes from D=3 to D=1. For a 2D Förster direct transfer process, we expect β = 1/3. This is close to our experimental value found for defects emitting in the 550–555 nm region (β = 0.32), but significantly larger than what is observed around the 568 nm emission wavelength. The model described by Equation (3) takes into account transfer via parallel channels to possibly all the defects of the distribution [49]. Limiting the transfer possibilities to only the nearest neighbors, examining the D=1 case (which can be solved analytically [49]), the relaxation function probability depends on $\beta_{NN}=1/7$∼0.14 [49] instead of β=1/6∼0.17, i.e., a 20% decrease. We can speculate that the stretching exponent in the 2D case also decreases when only the nearest neighbors are taken into account. From these assumptions, we can conclude that a 2D Förster direct transfer diffusion process limited to the nearest neighbors could describe the dynamics of emission of the defects in the tail of the MAPbBr${}_{3}$ emission. Whereas, the diffusion of the electrons in traps that exhibit an emission close to band-edge seem to have access to further-away traps, since the value of β matches that of the 2D case with no diffusion restriction. One feature which is not observed in other low-temperature reports [17], and only in a few at room temperature [45], is the remaining of the fast component associated to the exciton in the emission tail. This is a signature of the emission of localized excitons confined in minima of the local potential fluctuations, previously observed at room temperature in MAPbBr${}_{3}$ [45]. We have repeated these experiments for different repetition rates, from 5 MHz to 100 kHz, keeping the average incident power constant. This enables us to vary the short-time density of photons absorbed per laser pulse by the MAPbBr${}_{3}$ thin film, $n_{i}$ (with i= 5, 1, 0.5 and 0.1 being the repetition rates in MHz), without changing the number of photons absorbed by the thin film over a longer time average. It can be calculated as follows:

(4) $$N_{i}=\frac{<P>}{E_{phot}\times f_{rep}}$$

(5) $$n_{i}=\alpha \frac{N_{i}}{V_{abs}}$$

where $N_{i}$ is the number of photons per laser pulse, *i* = 5, 1, 0.5 and 0.1 (after the laser repetition rate in MHz), <P> = 240 nW/μm${}^{2}$ the average power onto the MAPbBr${}_{3}$ thin film, $E_{phot}=$ 2.8 eV is the energy of a laser photon, $f_{rep}$ the laser repetition rate (5, 1, 0.5 or 0.1 MHz), α= 0.5 the fraction of light being absorbed by the MAPbBr${}_{3}$ thin film at λ = 445 nm [43], and $V_{abs}=10^{13}$ cm${}^{3}$ is the absorption volume of the incident laser light. This allowed us to have $n_{i}$ varying up to a factor of 50, by controlling the repetition rate from $f_{rep}$ = 5 MHz ($n_{5}$ = 5 × 10${}^{15}$ cm${}^{-3}$) down to $f_{rep}$ = 100 kHz ($n_{0.1}$ = 2.5 × 10${}^{17}$ cm${}^{-3}$). We present in Figure 5a (550–550 nm) and b (568 ± 5 nm) examples of decay curves obtained for different $n_{i}$. We see that while close to the band-edge, as $n_{i}$ is increased, the stretched exponential component almost disappears, at 568 nm it decreases drastically by more than one order of magnitude but still exists, and the decays seem to have the same shape between 500 and 100 kHz but differ slightly from that of 5 MHz. The behavior close to the band-edge implies a saturation of the Förster diffusion mechanism at those wavelengths, which is the signature of defects filled by the laser excitation at $n_{0.1}$. The second behavior at 568 nm points to a value of β that can vary. To characterize those two behaviors, we have performed FLIM experiments over Zone 2, at different value of $n_{i}$. We first focus on the potential variation of the stretching exponent β in Figure 6. A pixel in Figure 6a–d represents the value of β as a colorscale, obtained from fitting the decay curves of every pixel with Equation (2), for $n_{5}$, $n_{1}$, $n_{0.5}$ and $n_{0.1}$, respectively. From those figures, one can acknowledge the spatial dependence of β at the micrometer scale. We see that at 5 MHz, in Figure 6a, β has values larger than for the other smaller repetition rates, backing the qualitative assumption made for Figure 5. Its value varies from β= 0.2 up to β= 0.5 for a few points, but has the majority of the image close to β∼0.3, while for the other images, it seems to only vary with an amplitude of ±10%. Interestingly, the few blue regions (β∼0.2) in Figure 6a, which represent the minimum value for the images at 5 MHz, become red regions as the repetition frequency is decreased (i.e., $n_{i}$ is increased). However, those regions do not experience a large variation in the absolute value of β, which stays around 0.2–0.24 for the four values of $n_{i}$. As the traps are filled, however, the green and red regions of Figure 6a tend to converge to that same range, and they are those which experience the largest change in absolute value of β. In the framework of the Förster direct transfer model, we can conclude that at 5 MHz, i.e., $n_{5}$, some regions behave closer to a 3D Förster direct transfer diffusion (yellow to red pixels), while the rest (large majority of the image) are more compatible to the 2D model. At larger $n_{i}$ (smaller repetition rates, Figure 6b,c), the images are more compatible with a truncated 2D Förster direct transfer model, where only the nearest neighbors are available for diffusion. Thus, the diffusion mechanism depends on the relative population of the traps. This behavior is confirmed in Figure 6e, which presents the average value of β for the whole image at a particular $n_{i}$, exhibiting that β= 0.32 at 5 MHz and falls to β= 0.23–0.24 for images recorded at 1, 0.5 and 0.1 MHz. It is worth noting that the value of β saturates at large $n_{i}$.

#### 3.2.2. Evolution of the Average Lifetime

We now focus on the second property observed in Figure 5: the evolution of the relative importance of the stretched exponential component compared to the fast component, as a function of $n_{i}$, or the absorbed density of photons by the MAPbBr${}_{3}$ thin films. First, we study the evolution of the average photon arrival time, $<\tau_{g}>$ defined as follows [50]:

(6) $$N_{fast}=A_{1}\times \int_{0}^{\infty }e^{-\gamma_{1}\times t}\;dt=\frac{A_{1}}{\gamma_{1}}=A_{1}\times <\tau_{fast}>$$

(7) $$N_{slow}=A_{2}\times \int_{0}^{\infty }e^{-{\left(\gamma_{2}\times t\right)}^{\beta }}\;dt=\frac{A_{2}}{\gamma_{2}\beta }\times \Gamma \left(1/\beta \right)=A_{2}\times <\tau_{slow}>$$

(8) $$<\tau_{g}>=\frac{N_{fast}<\tau_{fast}>+N_{slow}<\tau_{slow}>}{<N_{fast}>+<N_{slow}>}$$

where $N_{fast}$ and $N_{slow}$ are the number of photons in the fast and slow components, Γ(1/β) is the Gamma function evaluated at 1/β, and the other parameters are obtained from the fits and introduced in Equation (2). We have computed $<\tau_{g}>$ for the band-edge emission (550–555 nm) and the tail emission (568 ± 5 nm) over Zone 2 for $n_{5}$, $n_{0.5}$ and $n_{0.1}$. Results of that procedure are presented in Figure S5a–c (550–555 nm) and Figure S5d–f (568 ± 5 nm). The average lifetime $<\tau_{g}>$ of the emission closer to the band-edge shows little to no clear trend, but a slight acceleration at $n_{0.1}$ with $<\tau_{g}>$ comprised ∼50–100 ps, while evolving in the range ∼100–200 ps for smaller $n_{i}$. We saw, qualitatively, this behavior in Figure 5 where the stretched exponential component almost disappears from $n_{5}$ to $n_{0.1}$ for emission close to the band-edge. These results coupled to results from Figure S5a–c demonstrate that the weight of the fast component is dominating the decay at large $n_{i}$. Moreover, we can see that $<\tau_{g}>$ is spatially inhomogeneous and that the regions of longer lifetimes are the ones with smaller numbers of photons. In contrast, in the emission tail, Figure S5d–f exhibits a strong dependence of $<\tau_{g}>$ with $n_{i}$, going from dynamics ranging over $<\tau_{g}>∼$1–10 ns at $n_{5}$, down to $<\tau_{g}>∼$0.2–0.5 ns for $n_{0.1}$. The spatial variation of $<\tau_{g}>$ at the emission tail of the MAPbBr${}_{3}$ is anti-correlated to that of the band-edge: $<\tau_{g}>$ is shorter in the region of lower signals. Finally, the dynamics of $<\tau_{g}>$ over Figure S5f in the emission tail are close to that of Figure S5c near the band-edge, meaning that for $n_{0.1}=2.5\times 10^{17}$ cm${}^{-3}$, the fast component dominates both decays. These results exhibit quantitatively the relative decrease in the stretched component with increasing $n_{i}$ and its spatial distribution.

#### 3.2.3. Determination of the Density of Traps

We can go further in the analysis by focusing on the ratio

(9) $$\eta =\frac{N_{fast}}{N_{slow}}$$

**Figure 7:**
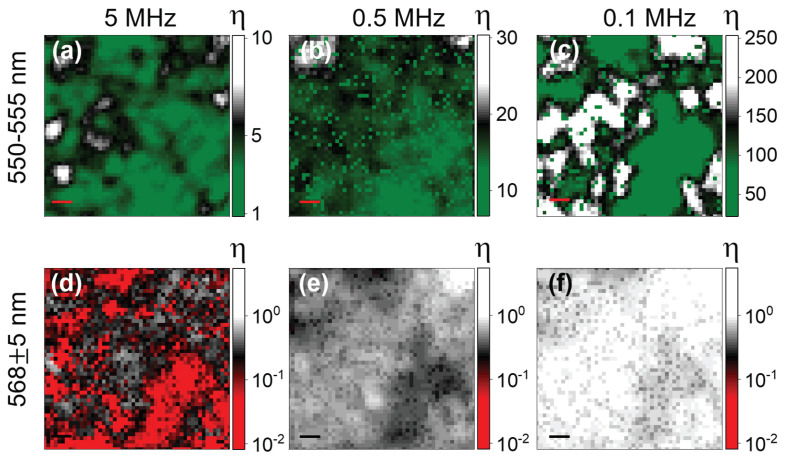
9 × 9 μm${}^{2}$, 50 × 50 pixel image of Zone 2 where a pixel takes the value calculated for η from Equation (9) represented as a colorscale. (**a**–**c**) Obtained from DET1 (550–555 nm) for repetition rates 5 MHz ((**a**), $n_{5}$ = 5 × 10${}^{15}$ cm${}^{-3}$), 500 kHz ((**b**), $n_{0.5}$ = 5 × 10${}^{16}$ cm${}^{-3}$) and 100 kHz ((**c**), $n_{0.1}$ = 2.5 × 10${}^{17}$ cm${}^{-3}$). (**d**–**f**) The corresponding values of η obtained for DET2 (568 ± 5 nm).

We present in Figure 7 an image of 9 × 9 μm${}^{2}$, 50 × 50 pixel of Zone 2 where η is represented as a colorscale. Figure 7a–c is obtained close to the band-edge emission (550–555 nm) for repetition rates 5 MHz ((a), $n_{5}$ = 5 × 10${}^{15}$ cm${}^{-3}$), 500 kHz ((b), $n_{0.5}$ = 5 × 10${}^{16}$ cm${}^{-3}$) and 100 kHz ((c), $n_{0.1}$ = 2.5 × 10${}^{17}$ cm${}^{-3}$). Figure 7d–f shows the corresponding values of η obtained for the tail if the emission (568 ± 5 nm). We can discriminate three cases for our interpretation: η≪1 where the stretched dynamics (emission driven by electron diffusion) dominates, η≫1 where the fast component (emission driven by excitons) and η∼1 where both contributions are comparable. At $n_{5}$ close to the band-edge, Figure 7a, most of Zone 2 emission looks comparable, at least with the same order of magnitude contribution of both excitons and emission arising from the electron diffusion process. There is η∼1–3 for most of the image, with a minority micron-sized region where η≫1, confirming that the emission dynamics are spatially dependent at the micrometer scale. In the tail of the emission, Figure 7d, the whole image presents η≪1, with a spatial modulation that shows η variation over one order of magnitude. In the red regions of Figure 7d, the emission due to the electron diffusion in the traps represents up to 99% of the emitted photons, i.e., virtually no contribution from excitons. As $n_{i}$ is increased, we observe a clear domination of exciton emissions at the band-edge, to finally obtain η∼30–250 depending on the region in Zone 2, Figure 7c. We thus conclude that more than 99.5% of the emission is from excitons at the band-edge for some regions of Zone 2 at $n_{0.1}=$ 2.5 × 10${}^{17}$ cm${}^{-3}$, while its weight was comparable to the emission due to Förster transfer at $n_{5}=5$× 10${}^{15}$ cm${}^{-3}$. The Förster direct transfer emission has thus been reduced by almost three orders of magnitude, relative to the exciton emission. This clearly points, coupled with the results obtained from CW experiments, to a saturation of the trap population close to the band-edge for $n_{0.1}$. The trend is similar at 568 nm and $n_{0.1}$, Figure 7e,f, where η∼10 in some regions, and excitons’ emission represents up to 90% of the emitted photons, while it was around 1% for $n_{5}$. We can thus be more specific combining the time-resolved and CW experiments and give an estimation of the population of traps in MAPbBr${}_{3}$ thin films prepared via the anti-solvent method at *n*∼10${}^{17}$ cm${}^{-3}$, which is two orders of magnitude larger than for a single crystal of MAPbBr${}_{3}$ [16].

## 4. Conclusions

To conclude, hyperspectral microscopy over 5 decades of excitation intensities performed on a MAPbBr${}_{3}$ thin film at T = 4.7 K revealed the spatial inhomogeneity of the emission wavelength, with micrometer-scale regions dominated by excitons (band-edge emission) or defects (red emission tail). Two-color, time-resolved experiments revealed a complex dynamic composed by the exciton (fast mono-exponential process ∼100–200 ps) and trap emissions (stretched exponential). The stretched exponential corresponds to a resonant Förster transfer in two dimensions in a randomly distributed ensemble of traps, with the electron diffusing through the traps and recombining with immobile acceptors. Varying the density of absorbed photons $n_{i}$ by the MAPbBr${}_{3}$ thin film, we find that the diffusion process shifts from a model where all the traps in the distribution are accessible for diffusion (low $n_{i}$) to a situation better described when only the nearest neighbors are available for the electron diffusion process (high $n_{i}$). We identified the spatial dependence of the stretching parameter β determining the dimensionality of the diffusion process. Tracking the evolution of the average emission lifetime $<\tau_{g}>$ as a function of $n_{i}$ revealed its spatial inhomogeneities at the micrometer scale. Finally, we found that as $n_{i}$ was increased, the relative number of photons in the fast component dramatically increased to reach more than 99.5% of the emission close to the band-edge at $n_{0.1}$ = 2.5 × 10${}^{17}$ cm${}^{-3}$, indicating a saturation of the emission from traps. This enabled us to estimate the concentration of available diffusing traps to *n*∼10${}^{17}$ cm${}^{-3}$ in MAPbBr${}_{3}$ thin films prepared via the anti-solvent method.

## Data Availability

Data are available on request from the authors.

## Supplementary Materials

The following supporting information can be downloaded at https://www.mdpi.com/article/10.3390/nano13162376/s1, Figure S1: XRD of the MAPbBr${}_{3}$ thin film; Figure S2: Low-temperature microscope set-up; Figure S3: Detailed hyperpsctral images of Zone1; Figure S4: IRF; Figure S5: FLIM data; Videos S1–S6: hyperspectral movies of Zone1 for intensities ranging from 1 nW/μm${}^{2}$ (S1) to 100 μW/μm${}^{2}$ (S6); Video S7: hyperspectral movie of *X*. (Reference [51] is cited in the Supplementary Materials).

## References

[1] Kim H.S., Lee C.R., Im J.H., Lee K.B., Moehl T., Marchioro A., Moon S.J., Humphry-Baker R., Yum J.H., Moser J.E. (2012). Lead Iodide Perovskite Sensitized All-Solid-State Submicron Thin Film Mesoscopic Solar Cell with Efficiency Exceeding 9%. Sci. Rep..

[2] Chung I., Lee B., He J., Chang R.P.H., Kanatzidis M.G. (2012). All-solid-state dye-sensitized solar cells with high efficiency. Nature.

[3] Lee M.M., Teuscher J., Miyasaka T., Murakami T.N., Snaith H.J. (2012). Efficient Hybrid Solar Cells Based on Meso-Superstructured Organometal Halide Perovskites. Science.

[4] Nayak P.K., Mahesh S., Snaith H.J., Cahen D. (2019). Photovoltaic Solar Cell Technologies: Analysing the State of the Art. Nat. Rev. Mater..

[5] Lan Y.F., Yao J.S., Yang J.N., Song Y.H., Ru X.C., Zhang Q., Feng L.Z., Chen T., Song K.H., Yao H.B. (2021). Spectrally Stable and Efficient Pure Red CsPbI _3_ Quantum Dot Light-Emitting Diodes Enabled by Sequential Ligand Post-Treatment Strategy. Nano Lett..

[6] Huang C.Y., Zou C., Mao C., Corp K.L., Yao Y.C., Lee Y.J., Schlenker C.W., Jen A.K.Y., Lin L.Y. (2017). CsPbBr _3_ Perovskite Quantum Dot Vertical Cavity Lasers with Low Threshold and High Stability. ACS Photonics.

[7] Fu C., Li Z.Y., Wang J., Zhang X., Liang F.X., Lin D.H., Shi X.F., Fang Q.L., Luo L.B. (2023). A Simple-Structured Perovskite Wavelength Sensor for Full-Color Imaging Application. Nano Lett..

[8] Zhou Y., Zhao H., Ma D., Rosei F. (2018). Harnessing the properties of colloidal quantum dots in luminescent solar concentrators. Chem. Soc. Rev..

[9] Chen H., Zhou L., Fang Z., Wang S., Yang T., Zhu L., Hou X., Wang H., Wang Z.L. (2021). Piezoelectric Nanogenerator Based on In Situ Growth All-Inorganic CsPbBr3 Perovskite Nanocrystals in PVDF Fibers with Long-Term Stability. Adv. Funct. Mater..

[10] Mykhaylyk V.B., Kraus H., Saliba M. (2019). Bright and Fast Scintillation of Organolead Perovskite MAPbBr_3_ at Low Temperatures. Mater. Horizons.

[11] Yu D., Wang P., Cao F., Gu Y., Liu J., Han Z., Huang B., Zou Y., Xu X., Zeng H. (2020). Two-dimensional halide perovskite as *β*-ray scintillator for nuclear radiation monitoring. Nat. Commun..

[12] Dong Q., Fang Y., Shao Y., Mulligan P., Qiu J., Cao L., Huang J. (2015). Electron-hole diffusion lengths >175 μm in solution-grown CH_3_NH_3_PbI_3_ single crystals. Science.

[13] Chen Z., Turedi B., Alsalloum A.Y., Yang C., Zheng X., Gereige I., AlSaggaf A., Mohammed O.F., Bakr O.M. (2019). Single-Crystal MAPbI_3_ Perovskite Solar Cells Exceeding 21% Power Conversion Efficiency. ACS Energy Lett..

[14] Leyden M.R., Meng L., Jiang Y., Ono L.K., Qiu L., Juarez-Perez E.J., Qin C., Adachi C., Qi Y. (2017). Methylammonium Lead Bromide Perovskite Light-Emitting Diodes by Chemical Vapor Deposition. J. Phys. Chem. Lett..

[15] Xu Z., Zeng Y., Meng F., Gao S., Fan S., Liu Y., Zhang Y., Wageh S., Al-Ghamdi A.A., Xiao J. (2022). A High-Performance Self-Powered Photodetector Based on MAPbBr_3_ Single Crystal Thin Film/MoS2 Vertical Van Der Waals Heterostructure. Adv. Mater. Interfaces.

[16] Wenger B., Nayak P.K., Wen X., Kesava S.V., Noel N.K., Snaith H.J. (2017). Consolidation of the optoelectronic properties of CH_3_NH_3_PbBr_3_ perovskite single crystals. Nat. Commun..

[17] Fu J., Jamaludin N.F., Wu B., Li M., Solanki A., Ng Y.F., Mhaisalkar S., Huan C.H.A., Sum T.C. (2019). Localized Traps Limited Recombination in Lead Bromide Perovskites. Adv. Energy Mater..

[18] Che X., Traore B., Katan C., Fang H.H., Loi M.A., Even J., Kepenekian M. (2019). Charge Trap Formation and Passivation in Methylammonium Lead Tribromide. J. Phys. Chem. C.

[19] Droseros N., Tsokkou D., Banerji N. (2020). Photophysics of Methylammonium Lead Tribromide Perovskite: Free Carriers, Excitons, and Sub-Bandgap States. Adv. Energy Mater..

[20] Niedzwiedzki D.M., Kouhnavard M., Diao Y., D’Arcy J.M., Biswas P. (2021). Spectroscopic investigations of electron and hole dynamics in MAPbBr_3_ perovskite film and carrier extraction to PEDOT hole transport layer. Phys. Chem. Chem. Phys..

[21] Gerhard M., Louis B., Frantsuzov P.A., Li J., Kiligaridis A., Hofkens J., Scheblykin I.G. (2021). Heterogeneities and Emissive Defects in MAPbI _3_ Perovskite Revealed by Spectrally Resolved Luminescence Blinking. Adv. Optical Mater..

[22] Saleh G., Biffi G., Di Stasio F., Martín-García B., Abdelhady A.L., Manna L., Krahne R., Artyukhin S. (2021). Methylammonium Governs Structural and Optical Properties of Hybrid Lead Halide Perovskites through Dynamic Hydrogen Bonding. Chem. Mater..

[23] Ventosinos F., Moeini A., Pérez-del Rey D., Bolink H.J., Schmidt J.A. (2022). Density of states within the bandgap of perovskite thin films studied using the moving grating technique. J. Chem. Phys..

[24] Xue H., Brocks G., Tao S. (2022). Intrinsic defects in primary halide perovskites: A first-principles study of the thermodynamic trends. Phys. Rev. Mater..

[25] Hu J., Zhang Y., Zhang X. (2022). Low-Temperature Discrimination of Defect States by Exciton Dynamics in Thin-Film MAPbBr_3_ Perovskite. J. Phys. Chem. Lett..

[26] de Quilettes D.W., Vorpahl S.M., Stranks S.D., Nagaoka H., Eperon G.E., Ziffer M.E., Snaith H.J., Ginger D.S. (2015). Impact of microstructure on local carrier lifetime in perovskite solar cells. Science.

[27] Moerman D., Eperon G.E., Precht J.T., Ginger D.S. (2017). Correlating Photoluminescence Heterogeneity with Local Electronic Properties in Methylammonium Lead Tribromide Perovskite Thin Films. Chem. Mater..

[28] Edri E., Kirmayer S., Henning A., Mukhopadhyay S., Gartsman K., Rosenwaks Y., Hodes G., Cahen D. (2014). Why Lead Methylammonium Tri-Iodide Perovskite-Based Solar Cells Require a Mesoporous Electron Transporting Scaffold (but Not Necessarily a Hole Conductor). Nano Lett..

[29] Yin W.J., Shi T., Yan Y. (2014). Unique Properties of Halide Perovskites as Possible Origins of the Superior Solar Cell Performance. Adv. Mater..

[30] de Quilettes D.W., Jariwala S., Burke S., Ziffer M.E., Wang J.T.W., Snaith H.J., Ginger D.S. (2017). Tracking Photoexcited Carriers in Hybrid Perovskite Semiconductors: Trap-Dominated Spatial Heterogeneity and Diffusion. ACS Nano.

[31] Phung N., Al-Ashouri A., Meloni S., Mattoni A., Albrecht S., Unger E.L., Merdasa A., Abate A. (2020). The Role of Grain Boundaries on Ionic Defect Migration in Metal Halide Perovskites. Adv. Energy Mater..

[32] Wang J., Zhang A., Yan J., Li D., Chen Y. (2017). Revealing the properties of defects formed by CH_3_NH_2_ molecules in organic-inorganic hybrid perovskite MAPbBr_3_. Appl. Phys. Lett..

[33] McGovern L., Koschany I., Grimaldi G., Muscarella L.A., Ehrler B. (2021). Grain Size Influences Activation Energy and Migration Pathways in MAPbBr_3_ Perovskite Solar Cells. J. Phys. Chem. Lett..

[34] Tilchin J., Dirin D.N., Maikov G.I., Sashchiuk A., Kovalenko M.V., Lifshitz E. (2016). Hydrogen-like Wannier–Mott Excitons in Single Crystal of Methylammonium Lead Bromide Perovskite. ACS Nano.

[35] Wang Q., Wu W. (2018). Temperature and excitation wavelength-dependent photoluminescence of CH3NH3PbBr3 crystal. Opt. Lett..

[36] Fang H., Duim H., Loi M.A. (2023). Disentangling Dual Emission Dynamics in Lead Bromide Perovskite. Adv. Opt. Mater..

[37] Dar M.I., Jacopin G., Meloni S., Mattoni A., Arora N., Boziki A., Zakeeruddin S.M., Rothlisberger U., Grätzel M. (2016). Origin of unusual bandgap shift and dual emission in organic-inorganic lead halide perovskites. Sci. Adv..

[38] Sarang S., Ishihara H., Chen Y.C., Lin O., Gopinathan A., Tung V.C., Ghosh S. (2016). Low temperature excitonic spectroscopy and dynamics as a probe of quality in hybrid perovskite thin films. Phys. Chem. Chem. Phys..

[39] Liu Y., Lu H., Niu J., Zhang H., Lou S., Gao C., Zhan Y., Zhang X., Jin Q., Zheng L. (2018). Temperature-dependent photoluminescence spectra and Decay dynamics of MAPbBr_3_ and MAPbI_3_ thin films. AIP Adv..

[40] Sadhukhan P., Pradhan A., Mukherjee S., Sengupta P., Roy A., Bhunia S., Das S. (2019). Low temperature excitonic spectroscopy study of mechano-synthesized hybrid perovskite. Appl. Phys. Lett..

[41] Shi J., Li Y., Wu J., Wu H., Luo Y., Li D., Jasieniak J.J., Meng Q. (2020). Exciton Character and High-Performance Stimulated Emission of Hybrid Lead Bromide Perovskite Polycrystalline Film. Adv. Opt. Mater..

[42] Wang K.H., Li L.C., Shellaiah M., Wen Sun K. (2017). Structural and Photophysical Properties of Methylammonium Lead Tribromide (MAPbBr_3_) Single Crystals. Sci. Rep..

[43] Baronnier J., Houel J., Dujardin C., Kulzer F., Mahler B. (2022). Doping MAPbBr_3_ hybrid perovskites with CdSe/CdZnS quantum dots: From emissive thin films to hybrid single-photon sources. Nanoscale.

[44] Shibata H., Sakai M., Yamada A., Matsubara K., Sakurai K., Tampo H., Ishizuka S., Kim K.K., Niki S. (2005). Excitation-Power Dependence of Free Exciton Photoluminescence of Semiconductors. Jpn. J. Appl. Phys..

[45] He H., Yu Q., Li H., Li J., Si J., Jin Y., Wang N., Wang J., He J., Wang X. (2016). Exciton localization in solution-processed organolead trihalide perovskites. Nat. Commun..

[46] Stranks S.D., Eperon G.E., Grancini G., Menelaou C., Alcocer M.J.P., Leijtens T., Herz L.M., Petrozza A., Snaith H.J. (2013). Electron-Hole Diffusion Lengths Exceeding 1 Micrometer in an Organometal Trihalide Perovskite Absorber. Science.

[47] Stranks S.D., Burlakov V.M., Leijtens T., Ball J.M., Goriely A., Snaith H.J. (2014). Recombination Kinetics in Organic-Inorganic Perovskites: Excitons, Free Charge, and Subgap States. Phys. Rev. Appl..

[48] Utzat H., Sun W., Kaplan A.E.K., Krieg F., Ginterseder M., Spokoyny B., Klein N.D., Shulenberger K.E., Perkinson C.F., Kovalenko M.V. (2019). Coherent single-photon emission from colloidal lead halide perovskite quantum dots. Science.

[49] Klafter J., Shlesinger M.F. (1986). On the relationship among three theories of relaxation in disordered systems. Proc. Natl. Acad. Sci. USA.

[50] Sillen A., Engelborghs Y. (1998). The Correct Use of “Average” Fluorescence Parameters. Photochem. Photobiol..

[51] Trinh A.L., Esposito A. (2021). Biochemical resolving power of fluorescence lifetime imaging: Untangling the roles of the instrument response function and photon-statistics. Biomed. Opt. Express.

