# Supplementary material for: Low-Temperature Emission Dynamics of Methylammonium Lead Bromide Hybrid Perovskite Thin Films at the Sub-Micrometer Scale
Source: Nanomaterials (Basel). 2023 Aug 19;13(16):2376. doi: 10.3390/nano13162376 (PMC10458237; doi:10.3390/nano13162376)
Supplement: Supplementary file 1 [file nanomaterials-13-02376-s001.zip › Houel_supplementary_revised_r2.pdf]

Supplementary Material for: Low-temperature emission dynamics of methylammonium lead bromide hybrid perovskite thin films at the sub-micrometer scale

Justine Baronnier, Benoît Mahler, Christophe Dujardin, and Julien Houel*

Institut Lumière-Matière, CNRS UMR5306, Université Lyon 1, Université de Lyon, 69622 Villeurbanne CEDEX, France

E-mail: julien.houel@univ-lyon1.fr

1. Structural characterization of the $MAPbBr_3$ thin film

We present in fig. S1 a X-ray diffraction diagram of our $MAPbBr_3$ crystalline thin film taken at T = 300 K in 2θ geometry. This XRD reveals a highly crystalline material with peaks corresponding to the (00X) directions of $MAPbBr_3$.[1] The fact the (00X) directions over-dominate the XRD signal is the signature of a highly oriented thin film.[2] The inset in fig. S1 presents a picture of the $MAPbBr_3$ thin films revealing a highly ransparent non-diffusive sample, signature of an homogeneous polycristalline thin film with sub-micrometer size domains.[2]

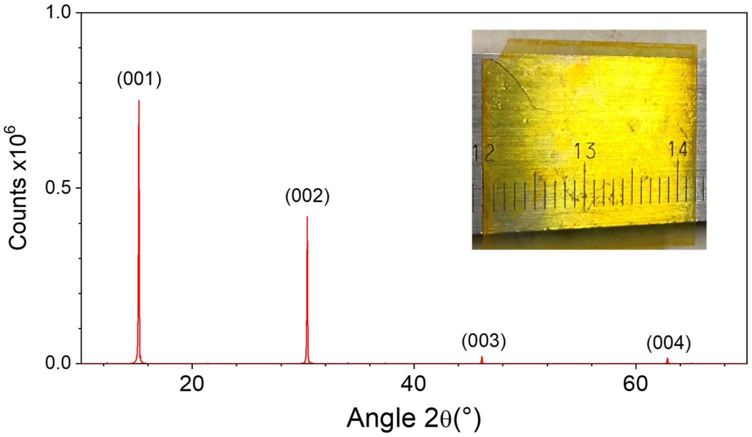

Figure S1: X-ray diffraction diagram of the $MAPbBr_3$ crystalline thin film at T = 300 K. Inset: picture of the transparent $MAPbBr_3$ thin film. Scale marks are centimeters, minor ticks are millimiters

2. Low-temperature experimental setup

We present in fig. S2 a detailed scheme of the low-temperature setup we used to performed the experiments presented in the main text. Excitation laser, either pulsed diode at λ =445 nm or CW at λ =532 nm, is coupled to a single mode fiber which delivers it to the microscope. The excitation light is passes through a first achromatic objective to generate a parallel beam. The beam is sent to a polarizer (Thorlabs, Glan-Thompson GTH10-A) and a half-waveplate (Thorlabs, AHWP05M-600) to maximize the reflection on the first polarizing beam splitter (Thorlabs, PBS101). The PBS reflects the beam to a quarter waveplate

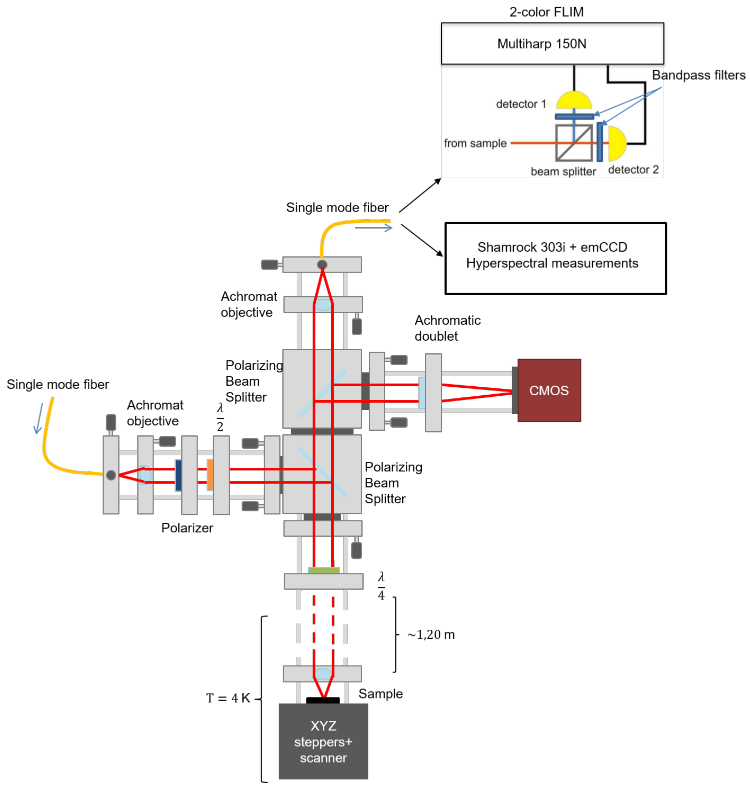

Figure S2: schematic view of the low-temperature experimental apparatus used to obtain the results presented in the main text

(Thorlabs, AQWP05M-600) which transforms the linear polarization into a circular one. That beam then enters the cryostat (Attocube, AttoDry1100) at T $=$ 4.8 K and travels down as a parallel beam to the microscope objective which focuses the excitation light onto the sample. The sample stands on a group of X, Y ans Z steppers (Attocube, ANPx51 and ANPz51) which allow for macroscopic (up to few mm) displacements with ~50-100 nm minimum step size. On top of the steppers, the sample is directly placed on an xyz piezo scanner (Attocube ANsX150/LT) which allows nm-size minimum step size, over a 9x9 μm^2 probing area. Light emitted by the sample is collected by the excitation objective and sent through the microscope as a parallel beam. It is transmitted by the two PBS to a focusing achromatic objective. Light is focused into a singe mode fiber, which directs the emission the the Handbury Brown and Twiss interferometer for two-color fluorescence lifetime imaging microscopy (FLIM), or to a spectrometer (Andor, Shamrock 303i) spectrometer coupled

to an emCCD camera (Andor, Newton 971) for hyperspectral measurements. Light was dispersed by a 600 l/mm grating allowing a spectral resolution of 0.24 nm. The HBT is composed by two single photon avalanche photodetectors (SPADs, Micron Photon Device, PDM series) coupled by a 50:50 beam splitter. In front of detector 1, we placed a longpass filter at 550 nm (Thorlabs, FELH0550) and a bandpass filter at 550±5 nm (Edmunds Optics), leading to a bandpass of 550-555 nm bandwidth. In front of detector 2, we placed a 568±5 nm (Edmunds Optics). The SPADs are connected to a multi-channel photon counting electronics (PicoQuant, MultiHarp 150N).

3. Hyperspcetral power dependence

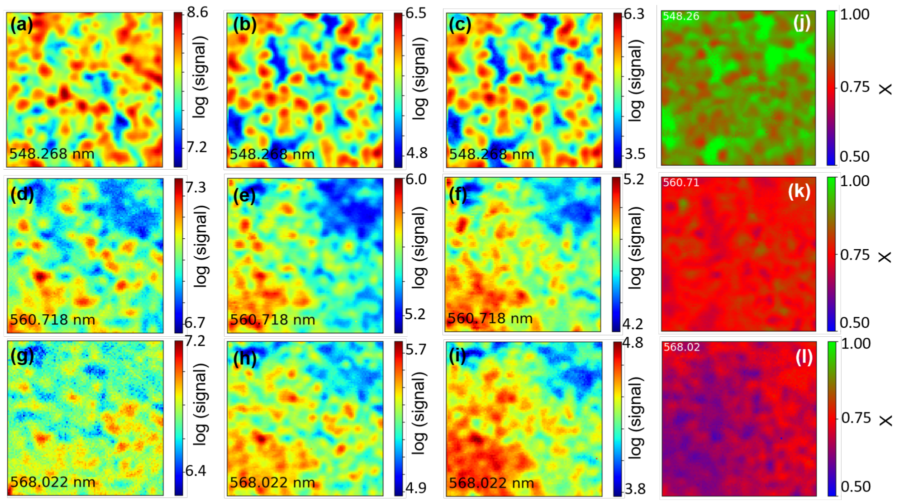

Figure S3: (a)-(i) Examples of images extracted from a 9x9 μm^2, 100x100 pixels hyperspectral imaging of Zone 1 in the $MAPbBr_3$ thin film for three different excitation intensities and emission wavelengths. (a)-(c) Spatially resolved emission signal of the $MAPbBr_3$ thin film at wavelength 548.3±0.40 nm for intensities of 100 $\mu W/\mu m^2$ (a), 1 $\mu W/\mu m^2$ (b) and 10 $nW/\mu m^2$ (c). (d)-(f) same as (a)-(c) but for emission spectra integrated over 560.7±0.40 nm. (g)-(i) same as (a)-(c) but for emission spectra integrated over 568±0.40 nm. (j)-(l) Image of 9x9 μm^2, 100x100 pixels representing X as a colorscale obtained from the fitting procedure (following eq. 1 from the main text) performed for every pixels of Zone 1 for 6 excitation intensities ranging over 5 decades from 100 $\mu W/\mu m^2$ down to 1 $nW/\mu m^2$.

4. Fluorescence lifetime imaging microscopy (FLIM) setup: instrument response function and count rate limitations

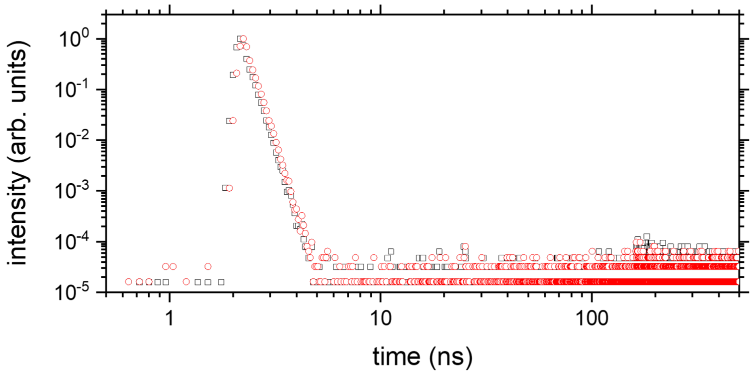

Figure S4: Temporal instrument response function (IRF) for the two channels of the FLIM setup.

We present in fig. S4 the typical instrument response function (IRF) of the FLIM setup used for the reconvolution process for data fitting. Note the rebound at t = 160 ns, which is seen in decay curves presented in the main text and is a characteristic of the MulitHarp 150N multichannel counter with a dead time set at 160 ns. Both channels exhibit a 3-points full-width at half maximum (FWHM), i.e. FWHM = 240 ps. For the fit, the shortest allowed decay rate was set at 50 ns^{-1}, considering our IRF and the number of counts in the histograms, following the recommendations in ref.[3] Since we are using a multi-channel electronics, the maximum count rate is not limited to 1-5 % of the repetition rate, but limited by the longest dead time, here the 160 ns of the MultiHarp 150N, equivalently 6.25 MHz. The sum of the photon count rate over the two channels was systematically checked and kept below ∼2 % of that frequency. To avoid fast-time pile-up effects, a statistical average of ∼1 photon/laser-cycle at maximum was observed, corresponding to a maximum combined count rate of ∼100 kHz.

5. Fluorescence lifetime imaging microscopy (FLIM) images

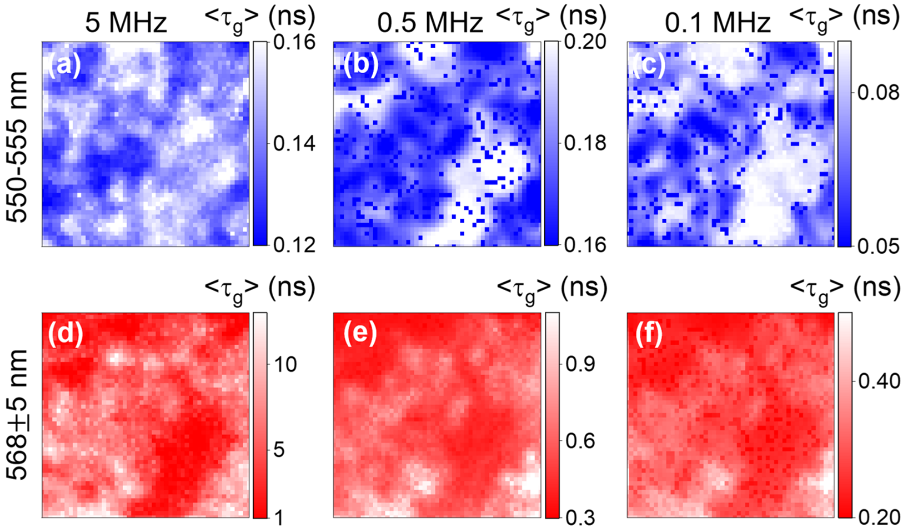

Figure S5: 9x9 μm^2, 50x50 pixels image of Zone 2 where a pixel takes the value calculated for $< \tau_g >$ from eq. 4c (in the main text) represented as a colorscale. (a)-(c) are obtained from DET1 (550-555 nm) for repetition rates 5 MHz ((a), $n_5 = 5\times10^{15}\,cm^{-3}$), 500 kHz ((b), $n_{0.5} = 5\times10^{16}\,cm^{-3}$) and 100 kHz ((c), $n_{0.1} = 2.5\times10^{17}\,cm^{-3}$). (d)-(f) are the corresponding values of $< \tau_g >$ obtained for DET2 (568±5 nm). The few full-blue pixels in the white regions of (b)-(c) are pixels where the automatic fitting procedure failed to converge correctly. They do not represent more than 10 % of the images.

References

1. Wang, K.-H.; Li, L.-C.; Shellaiah, M.; Wen Sun, K. Structural and Photophysical Properties of Methylammonium Lead Tribromide (MAPbBr3) Single Crystals. *Sci Rep* **2017**, *7*, 13643.

2. Baronnier, J.; Houel, J.; Dujardin, C.; Kulzer, F.; Mahler, B. Doping MAPbBr $_3$ hybrid perovskites with CdSe/CdZnS quantum dots: from emissive thin films to hybrid single-photon sources. *Nanoscale* **2022**, *14*, 5769–5781.

3. Trinh, A. L.; Esposito, A. Biochemical resolving power of fluorescence lifetime imaging: untangling the roles of the instrument response function and photon-statistics. *Biomed. Opt. Express* **2021**, *12*, 3775.
