# Supplementary figures and images for: Low-Temperature Emission Dynamics of Methylammonium Lead Bromide Hybrid Perovskite Thin Films at the Sub-Micrometer Scale
Source: Nanomaterials (Basel). 2023 Aug 19;13(16):2376. doi: 10.3390/nano13162376 (PMC10458237; doi:10.3390/nano13162376)

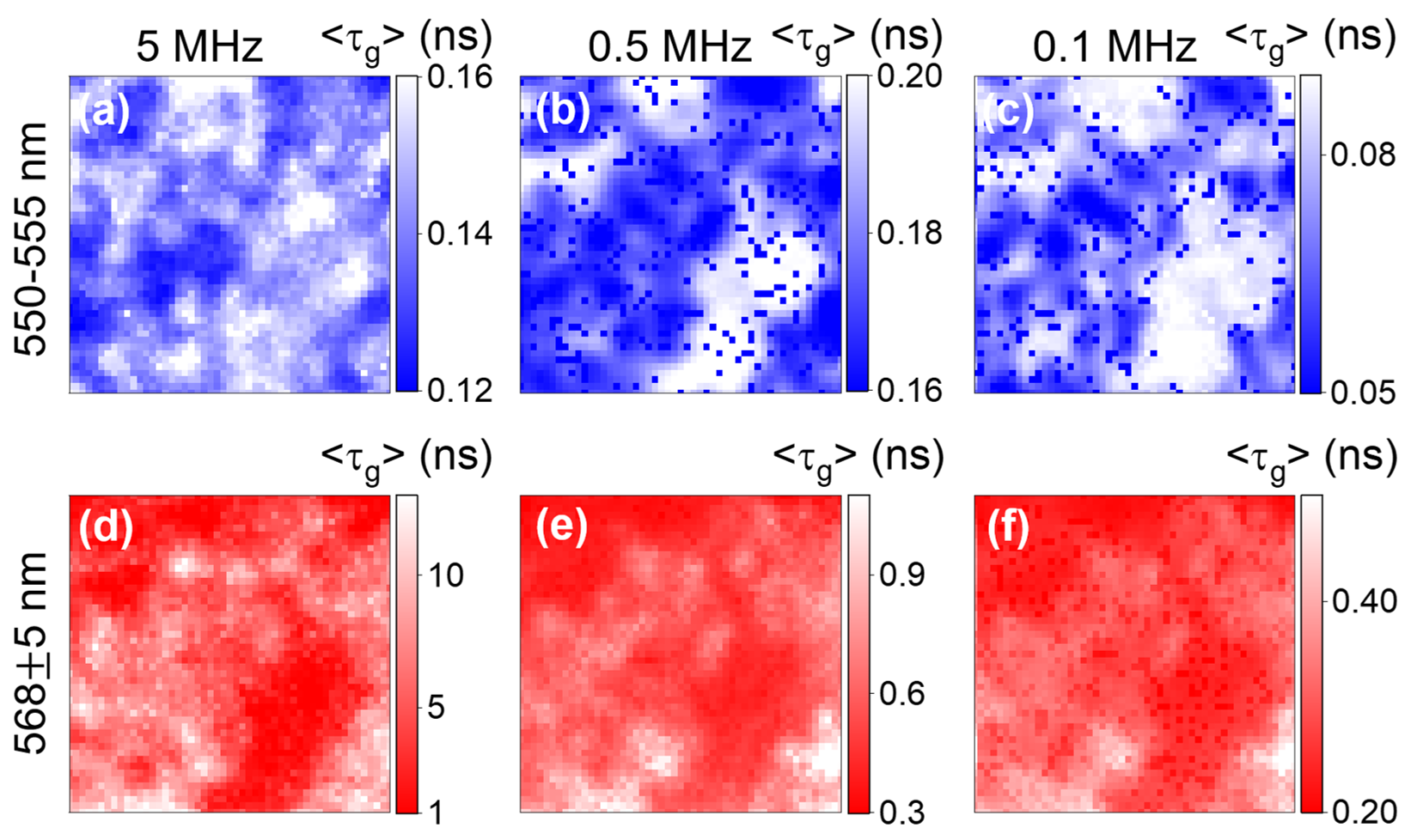

Supplement: Supplementary file 1 [file nanomaterials-13-02376-s001.zip › supp_FLIM.png]

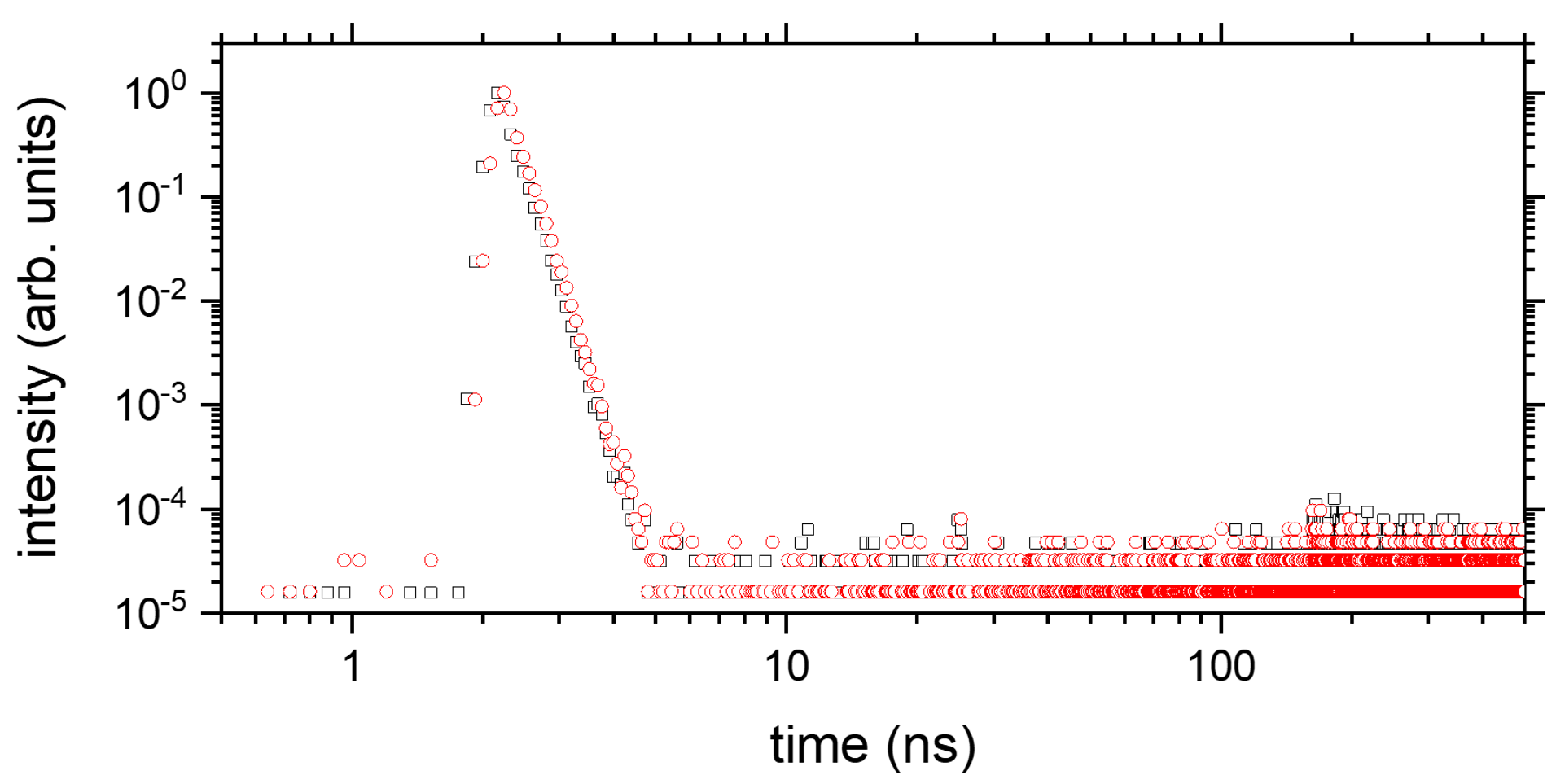

Supplement: Supplementary file 1 [file nanomaterials-13-02376-s001.zip › supp_IRFb.png]

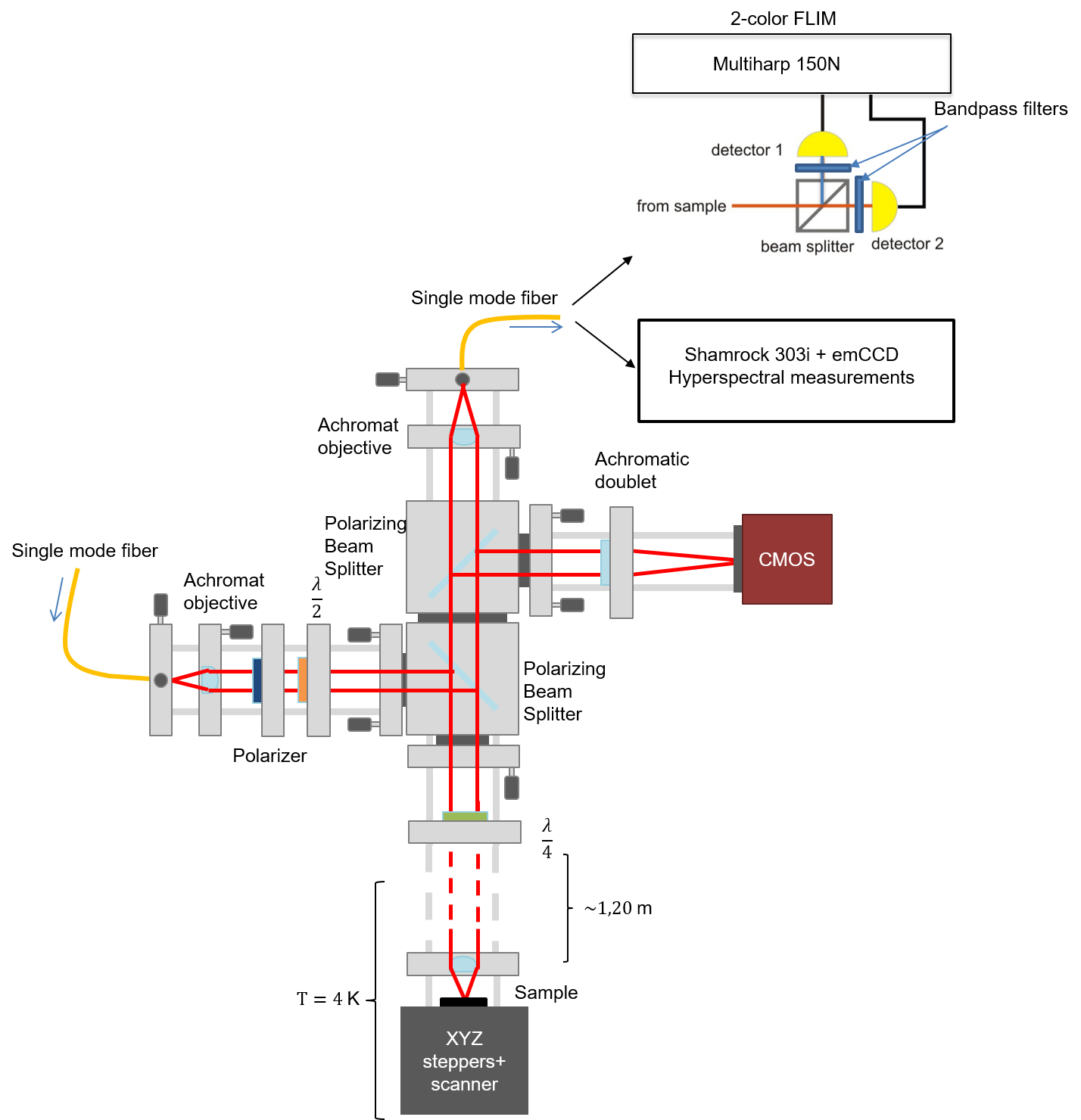

Supplement: Supplementary file 1 [file nanomaterials-13-02376-s001.zip › supp_lowT_uscope.png]

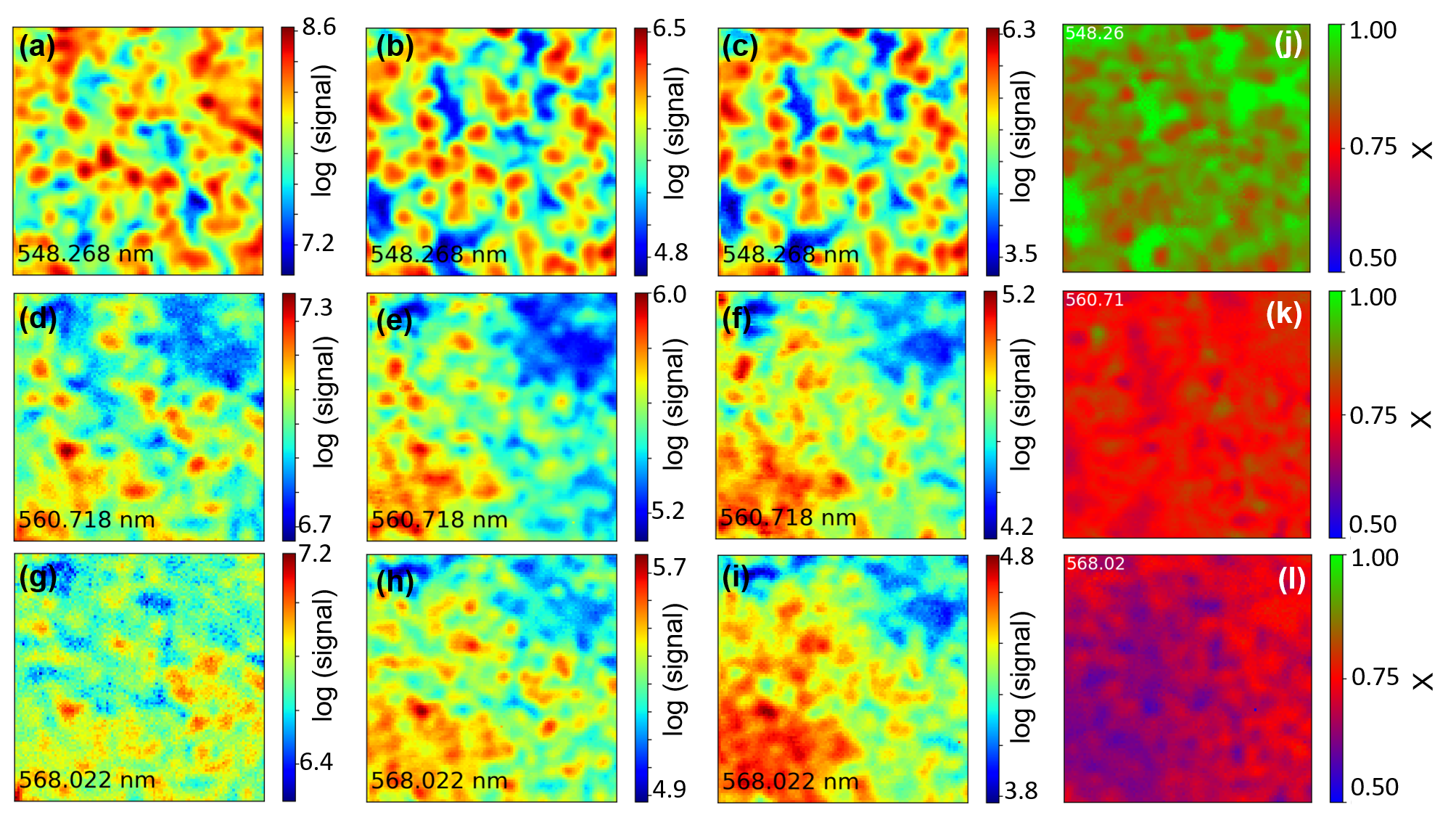

Supplement: Supplementary file 1 [file nanomaterials-13-02376-s001.zip › Supp_Xpower_dep.png]

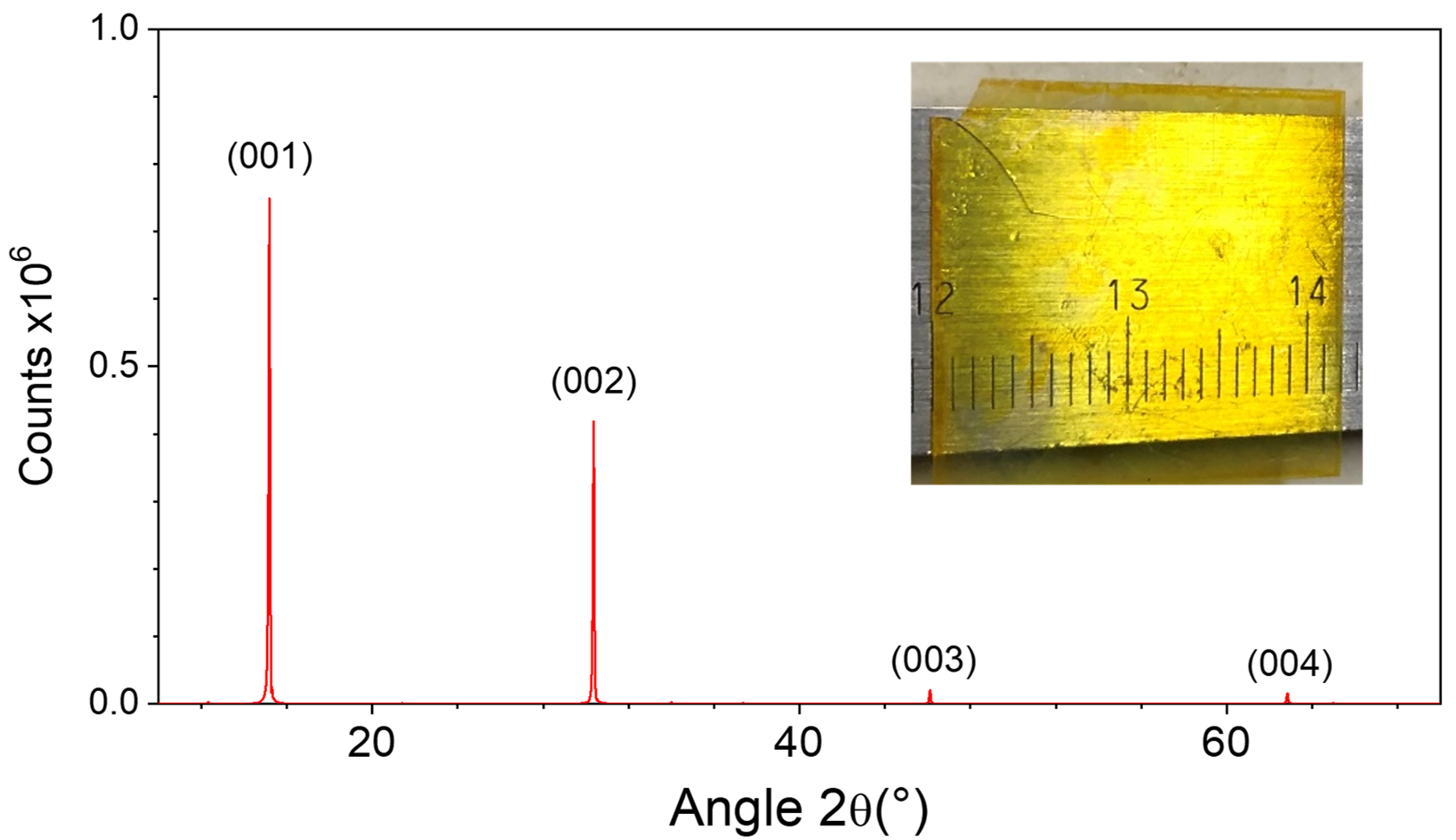

Supplement: Supplementary file 1 [file nanomaterials-13-02376-s001.zip › supp_xrd.png]
